# Supplementary material for: Inhibitory Effect and Mechanism of Dryocrassin ABBA Against Fusarium oxysporum
Source: Int J Mol Sci. 2025 Feb 13;26(4):1573. doi: 10.3390/ijms26041573 (PMC11855913; doi:10.3390/ijms26041573)
Supplement: Supplementary file 1 [file ijms-26-01573-s001.zip › Table S2. Dryocrassin ABBA had effects on various processes.pdf]

Table S2. Dryocrassin ABBA had effects on various processes.

	Gene id	log2FoldChange	*p*-Value	Product	Stat
HSP	FOXG_00233	−6.557428176	4.57×10^{-13}	30 kDa heat shock protein [Source:BROAD_F_oxysporum; Acc:FOXG_00233]	down
	FOXG_00378	−2.622230433	0.000330041	heat shock protein 78, mitochondrial precursor [Source:BROAD_F_oxysporum; Acc:FOXG_00378]	down
	FOXG_09418	−3.508647595	2.78×10^{-5}	heat shock protein HSP98 [Source:BROAD_F_oxysporum; Acc:FOXG_09418]	down
mfs	FOXG_09887	−2.381667113	0.000485036	hypothetical protein similar to major facilitator superfamily protein superfamily [Source:BROAD_F_oxysporum; Acc:FOXG_09887]	down
	FOXG_00029	1.47992372	4.79×10^{-10}	hypothetical protein similar to mfs amino acid transporter [Source:BROAD_F_oxysporum; Acc:FOXG_00029]	up
	FOXG_00479	1.033004122	0.004485202	hypothetical protein similar to MFS quinate transporter QutD [Source:BROAD_F_oxysporum; Acc:FOXG_00479]	up
	FOXG_02047	−6.856468946	0.000331397	hypothetical protein similar to mfs-multidrug-resistance transporter [Source:BROAD_F_oxysporum; Acc:FOXG_02047]	down
	FOXG_03950	−2.749470704	1.33×10^{-11}	hypothetical protein similar to MFS toxin efflux pump [Source:BROAD_F_oxysporum;Acc:FOXG_03950]	down
	FOXG_04943	9.588338475	6.41×10^{-11}	hypothetical protein similar to mfs myo-inositol transporter [Source:BROAD_F_oxysporum; Acc:FOXG_04943]	up
	FOXG_15236	−7.14892256	3.85×10^{-17}	hypothetical protein similar to mfs-multidrug-resistance transporter [Source:BROAD_F_oxysporum; Acc:FOXG_15236]	down
	FOXG_15681	2.796322541	2.58×10^{-5}	hypothetical protein similar to MFS peptide transporter [Source:BROAD_F_oxysporum; Acc:FOXG_15681]	up

	FOXG_17534	5.910328445	0.001304151	hypothetical protein similar to MFS sugar transporter [Source:BROAD_F_oxysporum; Acc:FOXG_17534]	up

Gene id	log2FoldChange	pvalue	product	stat	
FOXG_00233	-6.557428176	4.57E-13	30 kDa heat shock protein [Source:BROAD_F_oxysporum;Acc:FOXG_00233]	down	HSP
FOXG_00378	-2.622230433	0.000330041	heat shock protein 78, mitochondrial precursor [Source:BROAD_F_oxysporum;Acc:FOXG_00378]	down	
FOXG_09418	-3.508647595	2.78E-05	heat shock protein HSP98 [Source:BROAD_F_oxysporum;Acc:FOXG_09418]	down	
FOXG_09887	-2.381667113	0.000485036	hypothetical protein similar to major facilitator superfamily protein superfamily [Source:BROAD_F_oxysporum;Acc:FOXG_09887]	down	mfs
FOXG_00029	1.47992372	4.79E-10	hypothetical protein similar to MFS amino acid transporter [Source:BROAD_F_oxysporum;Acc:FOXG_00029]	up	
FOXG_00479	1.033004122	0.004485202	hypothetical protein similar to MFS quinate transporter QutD [Source:BROAD_F_oxysporum;Acc:FOXG_00479]	up	
FOXG_02047	-6.856468946	0.000331397	hypothetical protein similar to mfs-multidrug-resistance transporter [Source:BROAD_F_oxysporum;Acc:FOXG_02047]	down	
FOXG_03950	-2.749470704	1.33E-11	hypothetical protein similar to MFS toxin efflux pump [Source:BROAD_F_oxysporum;Acc:FOXG_03950]	down	
FOXG_04943	9.588338475	6.41E-11	hypothetical protein similar to MFS myo-inositol transporter [Source:BROAD_F_oxysporum;Acc:FOXG_04943]	up	
FOXG_09269	-2.999258742	3.04E-06	hypothetical protein similar to MFS drug transporter [Source:BROAD_F_oxysporum;Acc:FOXG_09269]	down	
FOXG_09811	-2.960490486	2.44E-05	hypothetical protein similar to MFS peptide transporter [Source:BROAD_F_oxysporum;Acc:FOXG_09811]	down	
FOXG_12240	-5.672136552	1.73E-17	hypothetical protein similar to mfs-multidrug-resistance transporter [Source:BROAD_F_oxysporum;Acc:FOXG_12240]	down	

FOXG_15236	-7.14892256	3.85E-17	hypothetical protein similar to mfs-multidrug-resistance transporter [Source:BROAD_F_oxysporum;Acc:FOXG_15236]	down	
FOXG_15681	2.796322541	2.58E-05	hypothetical protein similar to MFS peptide transporter [Source:BROAD_F_oxysporum;Acc:FOXG_15681]	up	
FOXG_17534	5.910328445	0.001304151	hypothetical protein similar to MFS sugar transporter [Source:BROAD_F_oxysporum;Acc:FOXG_17534]	up	
