# Supplementary material for: Inhibitory Effect and Mechanism of Dryocrassin ABBA Against Fusarium oxysporum
Source: Int J Mol Sci. 2025 Feb 13;26(4):1573. doi: 10.3390/ijms26041573 (PMC11855913; doi:10.3390/ijms26041573)
Supplement: Supplementary file 1 [file ijms-26-01573-s001.zip › Table S3. Primers used for real time PCR.pdf]

Table S3. Primers used for real time PCR.

	Gene id	Sequence forwards (5–3′)	Sequence reverse (5′-3′)
1	FOXG_12330	GTTCTCCTCACCAGTGTTCCAGATAGAC	GCCGTGACTCTGACTCCAACATG
2	FOXG_13051	ATGCGGACAGTCTTGACGGTGTT	ATGCTTTCGACGTTGGCTCCTCT
3	FOXG_02349	ACACCTTCGGCTTCGTCCAAACA	CTCACCAATGACAGCGAGGAACTTG
4	FOXG_08942	CGCGCTGACGTATCGAAGGACT	CCAAGACTTTCCCGATGCTCCCAT
5	FOXG_09571	GTCGCCGATACATCTCCTGCTCAT	GCTTGGTCCGTCTGTTGGTCCTAT
6	FOXG_16943	CGAGGACTTGTACCGCAAGGACAT	AGTAGGGAAAGCCGTTGGTGATGG
7	FOXG_13531	AGTTCGGATGACTGGGATGACACC	ACTGGCTTATCGCTTTACTGCTGGT
8	FOXG_15742	AAGACGACGGCAACTCCACCT	TTCCAACCCTTTCCACCAACAAAGT
9	FOXG_02047	GTCGAAATCTGTGTCGGAGAATGGC	GGCGGCCTTGATAGTCCTTCGTT
10	FOXG_04943	AATCACTGGTTTCTGGATGGCTTGC	TCTTCGGTGTGGATTCGGCAGAG
11	FOXG_17534	CGTACTCAGGTGGTACTTCCGACAA	TCTTCCTTGTGGCGCATGAGGT
12	actin	CGGTACTGGTGAGTTCGAGGCT	TGTTGATGGCGACAATGAGGTT
